# Quadruple Control Electrochromic Devices Utilizing Ce_4_W_9_O_33_ Electrodes for Visible and Near‐Infrared Transmission Intelligent Modulation

**DOI:** 10.1002/advs.202307223

**Published:** 2024-02-04

**Authors:** Dongyun Ma, Ting Yang, Xingzhe Feng, Pengfei Wang, Jiahui Huang, Jinmin Wang, Haizeng Li

**Affiliations:** ^1^ School of Materials and Chemistry University of Shanghai for Science and Technology Shanghai 200093 China; ^2^ Optics and Thermal Radiation Research Center, Institute of Frontier & Interdisciplinary Science Shandong University Qingdao Shandong 266237 China

**Keywords:** Ce_4_W_9_O_33_, electrochromic, energy storage, independent control, metal oxide

## Abstract

Electrochromic smart windows are promising for building energy savings due to their dynamic regulation of the solar spectrum. Restricted by materials or traditional complementary device configuration, precisely and independently controlling of visible (VIS) and near‐infrared (NIR) light is still on the drawing board. Herein, a novel Zn^2+^ electrochemically active Ce_4_W_9_O_33_ electrode is reported, which demonstrates three distinct states, including VIS and NIR transparent “bright and warm” state, VIS and NIR opaque “dark and cool” state, VIS transparent and NIR opaque “bright and cool” state. A dual‐operation mode electrochromic platform is also presented by integrating Ce_4_W_9_O_33_/NiO complementary device and Zn anode‐based electrochromic device (Ce_4_W_9_O_33_/Zn/NiO device). Such a platform enables an added VIS opaque and NIR transparent “dark and warm” state, thus realizing four color states through individually controlling Ce_4_W_9_O_33_ and NiO electrodes, respectively. These results present an effective approach for facilitating electrochromic windows more intelligent to weather/season conditions and personal preferences.

## Graphical abstract

A dual operation mode electrochromic platform, integrating Ce_4_W_9_O_33_‐NiO complementary device and zinc anode‐based electrochromic device (Ce_4_W_9_O_33_/Zn/NiO device), realizes four color states through individually controlling Ce_4_W_9_O_33_ and NiO electrodes, respectively.

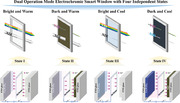

## 1 Introduction

Electrochromic smart windows, capable of dynamically tuning transmission upon applied external voltages,^[^ ^1^, ^2^, ^3^ ^]^ are promising for green buildings owing to their great significance in reducing building energy consumption through reductions in lighting, heating and cooling loads, etc.^[^ ^4^, ^5^, ^6^ ^]^ The integration of electrochromic characteristics and energy‐storage function in a single platform, namely an electrochromic energy‐storage device, is developed rapidly for energy‐saving and energy‐storage applications.^[^ ^7^, ^8^ ^]^ As a result, electrochromic batteries^[^ ^9^, ^10^, ^11^, ^12^, ^13^ ^]^ and electrochromic supercapacitors^[^ ^14^, ^15^, ^16^ ^]^ have received growing interest due to the potential to reduce the building energy consumption along with the storage of photovoltaic energy^[^ ^17^ ^]^ Unfortunately, although most state‐of‐the‐art electrochromic smart windows are capable of regulating visible (VIS) light and near‐infrared (NIR) light, the precise and independent control of VIS and NIR lights on demand through a single window is still undisclosed.

Recently, the localized surface plasmon resonance (LSPR) effect of electrochromic nanocrystals was reported as a promising strategy for selectively modulating VIS and NIR transmittance.^[^ ^18^, ^19^ ^]^ The LSPR is usually occurs in doped semiconductor nanocrystals, resulting in resonant absorption, scattering, and near field enhancement around the nanocrystals.^[^ ^20^ ^]^ This has spurred great interest in the synthesis of electrochromic metal oxide nanocrystals, from the electrochromic community, as these electrochromic metal oxide nanocrystals have tunable LSPR and thus resulting in a selective VIS and NIR regulation.^[^ ^21^ ^]^ For these oxide nanocrystals, the introduction of oxygen vacancies or dopants increases the density of free electrons and thus generating absorption in the NIR range due to the improved LSPR. Therefore, dual‐band electrochromic windows, capable of independently controlling VIS and NIR transmissions, are realized through utilizing these oxide nanocrystals.^[^ ^22^ ^]^ Nowadays, a series of pioneer metal oxide nanocrystals were designed for dual‐band electrochromic windows, which include TiO_2_@WO_3‐_ *_x_*,^[^ ^17^ ^]^ Nb‐TiO_2_ nanocrystals,^[^ ^21^ ^]^ Ta‐doped TiO_2_ nanocrystals,^[^ ^23^ ^]^ oxygen‐deficient TiO_2‐_ *_x_* nanocrystals,^[^ ^24^ ^]^ monoclinic WO_3‐_ *_x_* nanowires,^[^ ^25^, ^26^ ^]^ and Nb_12_O_29_ nanocrystals.^[^ ^27^ ^]^ Nonetheless, the LSPR of these nanocrystal films is often activated at high applied potentials, which brings challenges in addressing electrolyte decomposition issues. Furthermore, the complicated metal oxide nanocrystal synthesis and electrode fabrication bring strict limitations (e.g., shape/decay/size of nanocrystals, electrode fabrication method/annealing temperature/film thickness/interfacial engineering) to design high‐performance dual‐band electrochromic windows.

Additionally, most state‐of‐the‐art dual‐band electrochromic smart windows only present three color states, namely VIS and NIR transparent “bright and warm” state, VIS and NIR opaque “dark and cool” state, VIS transparent and NIR opaque “bright and cool” state,^[^ ^22^, ^28^, ^29^, ^30^ ^]^ which greatly restricts their applications in some situations requiring VIS opaque and NIR transparent. Although some attempts have demonstrated VIS opaque and NIR transparent state,^[^ ^31^, ^32^ ^]^ the inferior performance and incompatibility between separate VIS and NIR modulations of the complementary device is still a challenge to be resolved.

Nowadays, emerging Zn anode‐based electrochromic devices, a new and fundamentally different class of electrochromic devices, have attracted increasing attention due to their advantages including energy retrieval functionalities and device configuration flexibility.^[^ ^11^, ^13^, ^33^ ^]^ Such a unique configuration, sandwiching zinc frame within complementary electrochromic devices, enables independently controlling top and bottom electrochromic electrodes. Specifically, the top and bottom electrochromic electrodes can be independently addressed under the same or different redox states. As such, the color states can be highly broadened by separately tuning the color states of top and bottom electrochromic electrodes. Although we had reported that the employment of zinc anode‐based electrochromic platform enlarges the color palette within VIS light,^[^ ^17^, ^33^, ^34^ ^]^ the exploration of independently controlling of VIS and NIR transmission through zinc anode‐based electrochromic platform is unexploited due to the limitations of electrochromic oxide nanocrystals.

Herein, we demonstrate a dual‐band electrochromic device having four optical states by sandwiching a Zn frame metal anode within a Ce_4_W_9_O_33_/NiO complementary type device. The newly designed Ce_4_W_9_O_33_ film shows superior performance in comparison to recently reported electrochromic oxide nanocrystals due to its tunable electronic structure. It is envisioned that Ce doping can increase the amount of the vacancy in WO_3_ via the transformation of chemical valence (Ce^4+^→Ce^3+^),^[^ ^35^ ^]^ similar to the recently reported promising “bronze‐like” oxide structures,^[^ ^9^, ^36^, ^37^ ^]^ offering diverse optical properties due to the tunable electronic structure with varying cation intercalation.^[^ ^38^ ^]^ Such tunable electronic structure and vacancies introduced in the tungsten bronze‐like bimetallic oxide (i.e., Ce_4_W_9_O_33_) enables good electrochemical activity toward Zn^2+^ even when the sizes of the Ce_4_W_9_O_33_ particles are of the order of micrometers, which gets rid of the limitations occurs in designing nanocrystals. Therefore, the Zn^2+^‐triggered electrochromism of the Ce_4_W_9_O_33_ electrode, demonstrated for the first time, exhibits three optical state tunability due to the multistep electrochemical reactions (i.e., surface capacitance‐controlled electrochemical process and diffusion‐controlled electrochemical reaction process). With the Ce_4_W_9_O_33_ electrode carefully designed, we then established a dual operation mode electrochromic platform by integrating Ce_4_W_9_O_33_/NiO complementary device and zinc anode‐based electrochromic device (three‐electrodes Ce_4_W_9_O_33_/Zn/NiO device) for realizing four color states through individually controlling Ce_4_W_9_O_33_ and NiO electrodes. This exciting platform exhibits four states, including VIS‐NIR transparent “bright and warm” state, VIS‐NIR opaque “dark and cool” state, VIS transparent/NIR opaque “bright and cool” state, and an added VIS opaque/NIR transparent “dark and warm” state. This is the first demonstration that a single platform having precise and independent control of VIS and NIR transmission.

## 2 Results and Discussion

### 2.1 Preparation and Characterization of Ce_4_W_9_O_33_ Films

**Figure 1:**
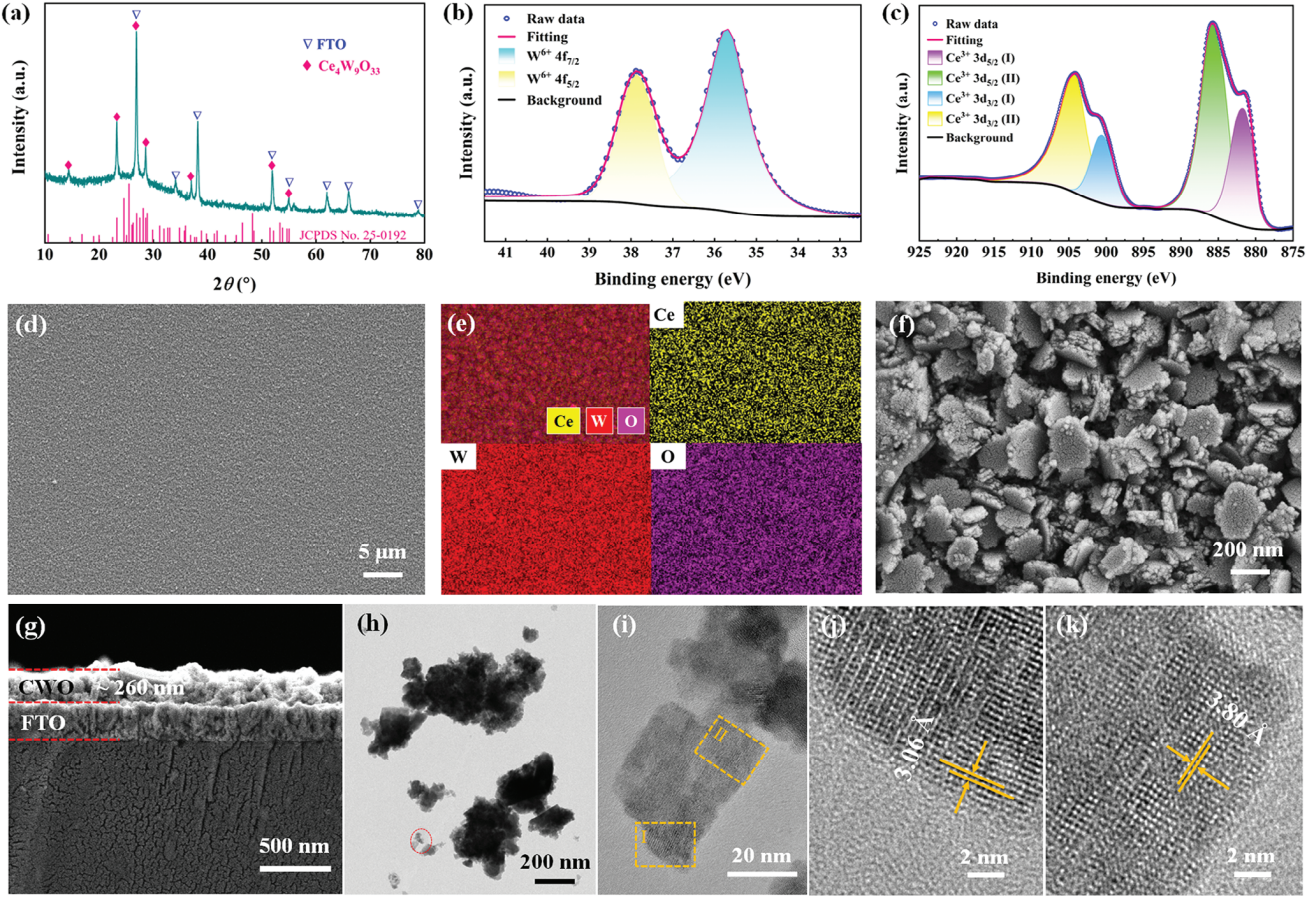
Structural characterizations of the Ce_4_W_9_O_33_ film. a) XRD pattern of the Ce_4_W_9_O_33_ film. XPS spectra of b) W 4f and c) Ce 3d for Ce_4_W_9_O_33_ film. d) Low magnification SEM image, e) corresponding energy dispersive spectroscopy (EDS) elemental mapping, f) high magnification SEM image, and g) cross‐sectional view of Ce_4_W_9_O_33_ film. h) TEM image of Ce_4_W_9_O_33_ scratched from the film, i) enlarged view of the marked region in (h), and j,k) HR‐TEM images of the marked region in (i).

The Ce_4_W_9_O_33_ film was directly grown on fluorine‐doped tin oxide (FTO) substrate using a hydrothermal method (see the Experimental Section). The growth of Ce_4_W_9_O_33_ films obeys a self‐seeded process,^[^ ^38^, ^39^ ^]^ which includes the nuclei formation and a subsequent growth of compact films. **Figure** **1**a depicts the X‐ray diffraction (XRD) pattern of the as‐grown film. Excluding the peaks of bare FTO glass (JCPDF 41–1445), the diffraction peaks at 2*θ* = 14.6, 23.3, 28.6, and 36.9° are readily assigned to a crystalline Ce_4_W_9_O_33_ phase (JCPDF 25–0192), which indicates that the Ce_4_W_9_O_33_ film is successfully coated onto FTO glass. The chemical states of the elements (Ce, W, and O) in the Ce_4_W_9_O_33_ film were investigated using X‐ray photoelectron spectroscopy (XPS, Figure S1, Supporting Information). The peaks at 37.8 and 35.7 eV, depicted in the XPS spectrum of W 4f (Figure 1b), correspond to the binding energies of W^6+^ 4f_5/2_ and 4f_7/2_ species, which reveals that W in the film is at its highest valence state. As is reported by Paparazzo and co‐authors,^[^ ^41^ ^]^ Ce 3d spectra can be deconvoluted into 10 peaks, including 4 peaks related to Ce^3+^ and 6 peaks related to Ce^4+^. The Ce 3d spectrum (Figure 1c) of Ce_4_W_9_O_33_ was fitted according to the above notation, where only the peaks of Ce^3+^ 3d_3/2_ and 3d_5/2_ species are observed. This indicates that Ce^3+^ are more stable in Ce_4_W_9_O_33_. This is in line with the previous results reported by Yoshimura and co‐authors,^[^ ^41^, ^42^ ^]^ which reveal that Ce^3+^ could exist stably in various Ce‐W‐O systems within Ce_4_W_9_O_33_ because W^6+^ strongly attracts O^2−^ from 2(CeO_2_) to stabilize Ce_2_O_3_.

Field‐emission scanning electron microscope (FESEM) image, depicted in Figure 1d, reveals that FTO glass is uniformly coated by Ce_4_W_9_O_33_ film. The uniform distributions of Ce, W, and O are observed in the corresponding energy dispersive spectroscopy (EDS) elemental mapping (Figure 1e) and scanning transmission electron microscopy (STEM)‐EDS elemental mapping (Figure S2, Supporting Information). High magnification SEM image reveals that the as‐prepared film is composed of irregular‐shaped blocks (Figure 1f), with a thickness of ≈260 nm (Figure 1g). Transmission electron microscopy (TEM) images, shown in Figure 1h,i further validate irregular‐shaped blocks of the Ce_4_W_9_O_33_ film. Selected‐area electron diffraction (SAED) pattern (Figure S3, Supporting Information) presents typical sharp spots from the Ce_4_W_9_O_33_ single‐crystal assemblies. Clear crystal lattice stripes, observed in the high‐resolution TEM (HR‐TEM) image (Figure 1j,k), affirm a high crystallinity of Ce_4_W_9_O_33_. The lattice spacings of I and II regions marked in Figure 1i correspond to the *d*‐spacings of the planes related to the diffraction peaks at 2*θ* = 28.6 and 23.3°, respectively. While Ce_4_W_9_O_33_ is a type of new tungsten bronze, further investigations, by the crystallographic structure community, are required to reveal detailed crystal‐plane indices of Ce_4_W_9_O_33_ in future studies.

### 2.2 Electrochemical Performance of the Ce_4_W_9_O_33_ Electrodes

**Figure 2:**
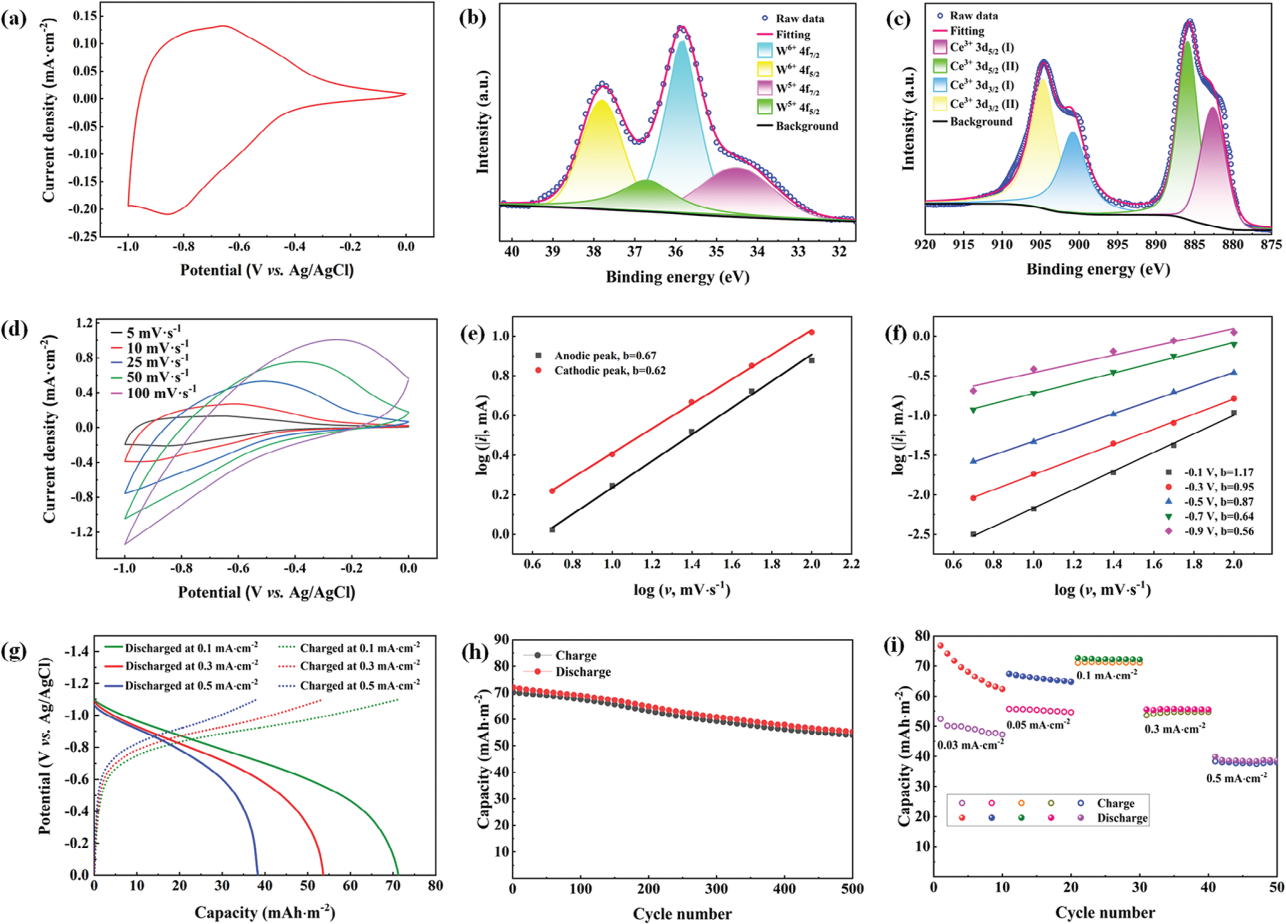
Electrochemical performance for the Ce_4_W_9_O_33_ electrode. a) C–V curve of the Ce_4_W_9_O_33_ electrode measured in 0.5 mol L^−1^ of ZnSO_4_ aqueous solution through a three‐electrode configuration. XPS spectra of b) W 4f and c) Ce 3d for the Ce_4_W_9_O_33_ film colored at −1.0 V. d) C–V curves at different scan rates. e) Plots of log*i* versus log*v* for Ce_4_W_9_O_33_ electrode, *i* is the peak current at different scan rates. f) Plots of log*i* versus log*v* for Ce_4_W_9_O_33_ electrode at different potentials. g) GCD curves at various current densities. h,i) Cycling performance of the Ce_4_W_9_O_33_ film electrode.

The electrochemical reactions and charge transfer kinetics for the Ce_4_W_9_O_33_ film were evaluated through a three‐electrode configuration using 0.5 mol L^−1^ of ZnSO_4_ as the electrolyte. **Figure** **2**a shows the cyclic voltammetry (CV) curve of the Ce_4_W_9_O_33_ electrode at a scan rate of 5 mV s^−1^, where the redox peaks ≈ −0.66/−0.86 V are attributed to redox reactions of Ce_4_W_9_O_33_, associated with Zn^2+^ intercalation and extraction. The large area enclosed by CV reveals the high electrochemical activity of Ce_4_W_9_O_33_ toward Zn^2+^, indicating that the Ce_4_W_9_O_33_ electrode is promising for Zn^2+^‐triggered electrochromism and zinc ion batteries. XPS analyses of the colored Ce_4_W_9_O_33_ electrode were performed to identify the Zn^2+^‐insertion reaction mechanisms. The results show that the intercalation of Zn^2+^ enables the reduction of W^6+^ to W^5+^ (Figure 2b), while the Ce remains in a Ce^3+^ state (Figure 2c). This points out that the electrochemical reactions and the corresponding coloration/bleaching of the Ce_4_W_9_O_33_ electrodes are induced by the redox reactions between W^6+^ and W^5+^. This is attributed to the higher redox potential of Ce^4+^/Ce^3+^ (1.44 V vs SHE) than W^6+^/W^5+^ (0.3 V vs SHE). The broadened peaks observed in the CV curves at various scan rates (Figure 2d) represent a typical intercalation pseudocapacitive behavior of Ce_4_W_9_O_33_.^[^ ^44^, ^45^, ^46^ ^]^ As it is well accepted that the charge storage in the CV curve is contributed from diffusion‐controlled electrochemical reactions and surface capacitance‐controlled processes,^[^ ^46^, ^47^ ^]^ we then investigated the two parts of capacitance contributions according to the following well‐established equations:^[^ ^48^, ^49^ ^]^

(1) $$i\;=\;av^{b}$$

(2) $$\log\left(i\right)=\;b\times log\left(v\right)+loga$$

where *a* and *b* are adjustable values, *i* and *v* are the current and scan rates, respectively. A *b*‐value of 0.5 represents that the current is a diffusion‐controlled one; a value of b = 1.0 indicates that the current is a surface‐controlled one, and a value of 0.5<b<1 means both diffusion‐controlled and surface‐controlled mechanisms are involved. Figure 2e demonstrates a plot of log(*i*) versus log(*ν*) from 5 to 100 mV s^−1^ for both anodic and cathodic peaks. The calculated *b*‐value of 0.67/0.62 implies that the reaction kinetics involve both surface‐controlled and diffusion‐controlled process. While electrochromic performance (i.e., the color/transmittance change) is often determined by the applied potentials, we then examined the *b*‐values using the current related to different potentials at various scan rates. Notably, the *b*‐value approaches 1.0 at −0.3/−0.4/−0.5 V, and is bigger than 1.0 at −0.1/−0.2 V, implying a surface capacitance‐controlled process. While the *b*‐values at −0.8, −0.9 and −1.0 V are close to 0.5 (Figure 2f; Figure S4, Supporting Information), indicating a diffusion‐dominated process. In this regard, the Ce_4_W_9_O_33_ electrodes are capable of showing various optical states by controlling different types of electrochemical reactions.

Additionally, we reveal that the Ce_4_W_9_O_33_ electrode has good galvanostatic charge‐discharge (GCD) characteristics (Figure 2g–i; Figure S5, Supporting Information). These results further affirm the great potential of Ce_4_W_9_O_33_ electrode for zinc ion batteries, which has not yet been reported to date. The charging and discharging areal capacities of the Ce_4_W_9_O_33_ electrode at 0.1 mA cm^−2^ are 71.5 and 71.2 mAh m^−2^, respectively. Such values are better than those of WO_3_ and WO_3_‐PEDOT in ZnSO_4_, LiClO_4,_ and H_2_SO_4_ electrolytes.^[^ ^11^, ^28^, ^50^ ^]^ The excellent rate performance of the Ce_4_W_9_O_33_ electrode (53.8% capacity retention at 0.5 mA cm^−2^) warrants the rapid electrochromic switching properties of the Ce_4_W_9_O_33_ electrode (Figure 2g). Moreover, during the charging process, the Ce_4_W_9_O_33_ electrode gradually changes its color from transparent to dark blue, accompanying the transmittance decrease from 88.0% to 11.9% at 633 nm (Figure S6, Supporting Information). This indicates that the areal capacity of the Ce_4_W_9_O_33_ electrode can be demonstrated from its color/transmittance, which is promise for visually displaying energy storage levels in real time. The Ce_4_W_9_O_33_ electrode also exhibits a good charging/discharging cycle performance at 0.1 mA cm^−2^, maintaining ≈80.0% of its initial value after 500 cycles (Figure 2h). The cycle stability deteriorates expectedly as the charging/discharging rates decrease, and improves with increased charging/discharging rates (Figure 2i). These results further confirm the Ce_4_W_9_O_33_ electrode's good compatibility for rapid electrochromic switching applications

### 2.3 Electrochromic Performance of the Ce_4_W_9_O_33_ Electrode

**Figure 3:**
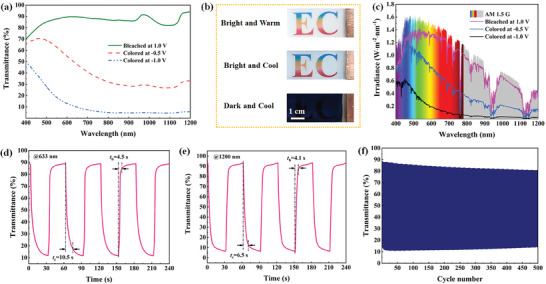
Electrochromic performance for the Ce_4_W_9_O_33_ electrode. a) Visible‐near infrared transmittance spectra of the Ce_4_W_9_O_33_ electrode at three different applied potentials. b) Digital photographs, and c) solar irradiance spectra of the Ce_4_W_9_O_33_ film electrode at different states. d) Real‐time transmittance changes during reversible switching measured at 633 nm and e) 1200 nm. f) Cycle performance of the Ce_4_W_9_O_33_ electrode.

A potential dependent electrochromic response is particularly anticipated for the independent control of VIS and NIR transmittance, making electrochromic technology more intelligent and efficient in saving a building's energy consumption. The electrolyte (i.e., 0.5 m ZnSO_4_ solution) used for investigating the electrochromic performance of the Ce_4_W_9_O_33_ electrode not only provides enough zinc cations for electrochemical redox reactions but also enables a reduced haze effect of the Ce_4_W_9_O_33_ electrode (Figure S7, Supporting Information). **Figure** **3**a shows the transmittance changes of the Ce_4_W_9_O_33_ electrode at three different applied potentials, and the results are summarized in **Table** **1**. The Ce_4_W_9_O_33_ electrode, being bleached at 1.0 V, is colorless and transparent at both VIS (85.8%) and NIR (85.8%) light region, which corresponds to the “bright and warm” state (Figure 3b). When a potential of −0.5 V is applied to the Ce_4_W_9_O_33_ electrode, the film is light blue in color. Such an electrode exhibits a “bright and cool” state, where 70.8% of the NIR light is blocked while maintaining a good transmittance within the VIS light region (62.8% at 550 nm). According to the above results and discussion (Figure 2f), this state, triggered by −0.5 V, is mostly related to the surface capacitance‐controlled electrochemical process. Such an electrochemical process is considered as a surface plasmon resonance (SPR) effect. That is, the NIR transmittance regulation reported in this work, originated from the SPR effect through surface‐controlled capacitive charging, is different from previously reported LSPR absorption.^[^ ^23^, ^51^ ^]^ Furthermore, a “dark and cool” state is activated by Zn^2+^ intercalation into the Ce_4_W_9_O_33_ at −1.0 V. In this state, 95.1% NIR light and 80.8% VIS light are blocked by the dark‐blue colored electrode, demonstrating a low transmittance of 11.7% in the solar spectrum range of 400–1200 nm. Since the Ce_4_W_9_O_33_ electrode is capable of regulating VIS and NIR lights at different optical states, the solar irradiance transmittances of the three color states were calculated for evaluating the actual solar irradiation modulation (Figure 3c). In the “bright and cool” state, the Ce_4_W_9_O_33_ electrode blocks 70.5% of the NIR radiation, while maintaining a transmittance of 55.9% in the VIS region. It is envisioned that almost all of the NIR radiation (95.2%) could be blocked at the “dark and cool” state, which is of great potential for reducing energy consumption through windows.

**Table 1:** Integrated optical transmittance (*T*) and solar irradiance transmittance (*T*′) for the Ce_4_W_9_O_33_ film and Ce_4_W_9_O_33_/Zn/NiO device in the VIS (400–780 nm), NIR (780–1200 nm) and whole solar (400–1200 nm) regions at different states.

Film/Device^a^ ^)^	State^b^ ^)^	*T*_VIS_ [%]	*T*_NIR_ [%]	*T*_sol_ [%]	$T_{\mathrm{VIS}}^{\prime }$ [%]	$T_{\mathrm{NIR}}^{\prime }$ [%]	$T_{\mathrm{sol}}^{\prime }$ [%]
Ce_4_W_9_O_33_ film	Bright and Warm	85.8	87.0	86.4	85.8	86.9	86.2
	Bright and Cool	54.9	29.2	41.4	55.9	29.5	46.9
	Dark and Cool	19.2	4.9	11.7	19.6	4.8	14.6
Ce_4_W_9_O_33_/Zn/NiO device	Bright and Warm	66.8	58.9	69.5	66.9	61.0	64.9
	Bright and Cool	40.0	19.7	29.4	40.8	20.7	33.9
	Dark and Warm	22.7	48.2	36.3	22.4	47.9	31.1
	Dark and Cool	7.3	4.0	5.6	7.4	4.1	6.2

^a)^ T=∫T(λ)dλ/∫dλ; ^b)^ $T^{\prime }=\int \varphi \left(\lambda \right)T\left(\lambda \right)d\lambda /\int \varphi \left(\lambda \right)d\lambda$, where *T*(*λ*) is the transmittance at a wavelength of *λ*, and *φ*(*λ*) is the solar irradiance at 1.5 air mass.

The switching times for the Ce_4_W_9_O_33_ electrode were investigated by the dynamic transmittance changes between different operating states. The switching speed between the “bright and warm” and “dark and cool” states was measured at 633 nm (Figure 3d), where the coloration and bleaching times are calculated to be 10.5 and 4.5 s, respectively. While a faster switching speed of 6.5/4.1 s for coloration/bleaching is achieved at 1200 nm (Figure 3e), as this process is related with surface capacitance‐controlled electrochemical process. Moreover, the Ce_4_W_9_O_33_ electrode delivers a high coloration efficiency of 98.3 cm^2^ C^−1^ at 1200 nm (Figure S8, Supporting Information), which is almost 1.5 times of the value at 633 nm (Figure S9, Supporting Information). These results, along with the transmittance modulation, reveal that Ce_4_W_9_O_33_ is an excellent cathodically coloring electrochromic material for effective regulation of VIS and NIR lights. Cycle stability is a very important metric for practical applications of electrochromic materials and devices. As described in Figure 3f, the Ce_4_W_9_O_33_ electrode demonstrates 500 reversible switching times between the “bright and warm” and “dark and cool” states, maintaining 88.9% of its initial optical modulation. Since this is the first demonstration of an electrochromic Ce_4_W_9_O_33_ electrode, studies on improving its cycling stability require further investigation in future research.

### 2.4 Electrochromic Performance of the Ce_4_W_9_O_33_/Zn/NiO Device

**Figure 4:**
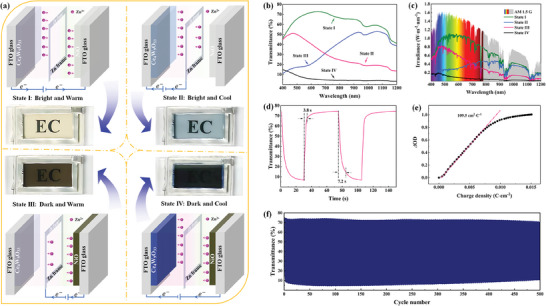
Structure and electrochromic performance of the Ce_4_W_9_O_33_/Zn/NiO device. a) Schematic illustration of the structure and working mechanism for the Ce_4_W_9_O_33_/Zn/NiO device. b) Visible‐near infrared transmittance, and c) solar irradiance spectra of the Ce_4_W_9_O_33_/Zn/NiO device. d) In situ transmittance variation, and e) optical density with respect to the charge density recorded at 633 nm when the device switched between State I and State IV. f) Cycle performance of the Ce_4_W_9_O_33_/Zn/NiO device measured between State I and State IV.

Previous studies regarding dual‐band electrochromism usually demonstrated three color states for adjusting the sunlight and radiation,^[^ ^27^, ^28^, ^29^ ^]^ which cannot meet the requirements of some situations (e.g., summer nights with large temperature difference between daytime and night time), where NIR transparent is very necessary to keep the indoor cool while VIS opaque for indoor privacy. In this regard, we constructed a dual‐mode electrochromic platform by integrating zinc anode‐based and complementary electrochromic devices to realize the independent regulation of VIS and NIR lights. The dual‐mode electrochromic platform, illustrated in **Figure** **4**a, is constructed by sandwiching a zinc frame within the Ce_4_W_9_O_33_ electrode and NiO electrode. Here, the Zn^2+^‐triggered electrochromic performance of the NiO electrode is revealed for the first time. As shown in Figure S10 (Supporting Information), the NiO electrode exhibits a large Zn^2+^‐triggered optical modulation in the VIS light region (66.6% at 550 nm). The optical modulation of NiO, larger than those of previously reported NiO electrodes in alkaline electrolytes,^[^ ^52^, ^53^ ^]^ is attributed to the porous nanoflake structure of the electrode (Figure S11, Supporting Information). Furthermore, the NiO electrode is colored when being oxidized, which is a perfect candidate for serving as counter electrode coupling with a cathodically coloring electrochromic electrode within a complementary device. Therefore, such NiO electrode is selected in the dual‐mode electrochromic platform to match the charge storage capability with the Ce_4_W_9_O_33_ electrode (Figure S12, Supporting Information), which enables the device to work more efficiently. The dual‐mode Ce_4_W_9_O_33_/Zn/NiO electrochromic platform could realize four color states through a pairwise combination among the three electrodes. When a voltage of 2.0 V is applied between the Ce_4_W_9_O_33_ working electrode and NiO counter electrode, the device achieves a “bright and warm” state, which allows both VIS and NIR lights transmitting through the window. The reaction mechanism refers to the oxidation of the Ce_4_W_9_O_33_ electrode and reduction of the NiO electrode, along with the extraction and insertion of Zn^2+^ ions, respectively. The “bright and cool” state is triggered by applying a voltage of 0.3 V between the Ce_4_W_9_O_33_ (cathode) and Zn electrodes (anode). The light blue‐colored device (Figure 4a) blocks 80.3% (Figure 4b) of sunlight and 79.3% (Figure 4c) of solar irradiation in the NIR region while maintaining a transmittance of 45.8% at 550 nm. Notably, the Ce_4_W_9_O_33_ electrode can also be self‐colored by connecting it to the Zn electrode (Figure S13a, Supporting Information) with a self‐coloring speed of ≈12.8 s (Figure S14a, Supporting Information). Similarly, the Ce_4_W_9_O_33_ electrode can be fully bleached by applying a 1.5 V bias between Ce_4_W_9_O_33_ and Zn electrodes (Figure S13b, Supporting Information), allowing the device back to the “bright and warm” state. When a bias of 2.0 V is applied between the Zn (cathode) and NiO (anode) electrodes, the device could switch to a “dark and warm” state. In this state, the NiO electrode is colored, and the optical modulation of the device reaches to 52.0% at 550 nm but having a good transmittance for both sunlight (48.2%) and solar irradiation (47.9%) in the NIR light region (Figure 4b,c and **Table** **1**). The optical modulation is larger than the reported WO_3_ dual‐band device^[^ ^32^ ^]^ and PANI/ITO electrode^[^ ^32^ ^]^ in dark states. The device can recover to the “bight and warm” state by connecting NiO and Zn electrodes (Figure S13c, Supporting Information). The self‐bleaching process takes only 5.8 s from the colored state to the bleached state (Figure S14b, Supporting Information). Furthermore, the “dark and cool” state can be realized by applying a voltage of −2.0 V between the Ce_4_W_9_O_33_ and NiO electrodes. Almost all of the VIS (92.7%) and NIR (96.0%) lights are blocked at this “dark and cool” state and only allow 5.6% of sunlight and 6.2% of solar irradiation transmission. The above four color states shown in a single platform (i.e., Ce_4_W_9_O_33_/Zn/NiO device), demonstrated for the first time, enable a more intelligent, efficient, and independent modulation of VIS and NIR lights.

Benefiting from the fast‐switching speed of the NiO electrode (Figure S15, Supporting Information), the Ce_4_W_9_O_33_/NiO complementary device delivers faster coloration/bleaching times of 7.2/3.8 s (Figure 4d) between the “bright and warm” and “dark and cool” states. Moreover, the Ce_4_W_9_O_33_/NiO complementary device shows a high coloration efficiency of 109.5 cm^2^ C^−1^ (Figure 4e), which is higher than those of both single Ce_4_W_9_O_33_ and NiO electrodes (Figure S16, Supporting Information). Besides, the Ce_4_W_9_O_33_/NiO complementary device demonstrates a higher reversibility (Figure 4f) than those of both single Ce_4_W_9_O_33_ (Figure 3f) and NiO electrodes (Figure S17, Supporting Information), maintaining 91.2% of its initial optical modulation after 500 reversible switching times. These excellent results reveal that the NiO electrode employed in this work has good compatibility with the Ce_4_W_9_O_33_ electrode for constructing a complementary electrochromic device.

### 2.5 Energy Retrieval Functionality of the Ce_4_W_9_O_33_/Zn/NiO Device

**Figure 5:**
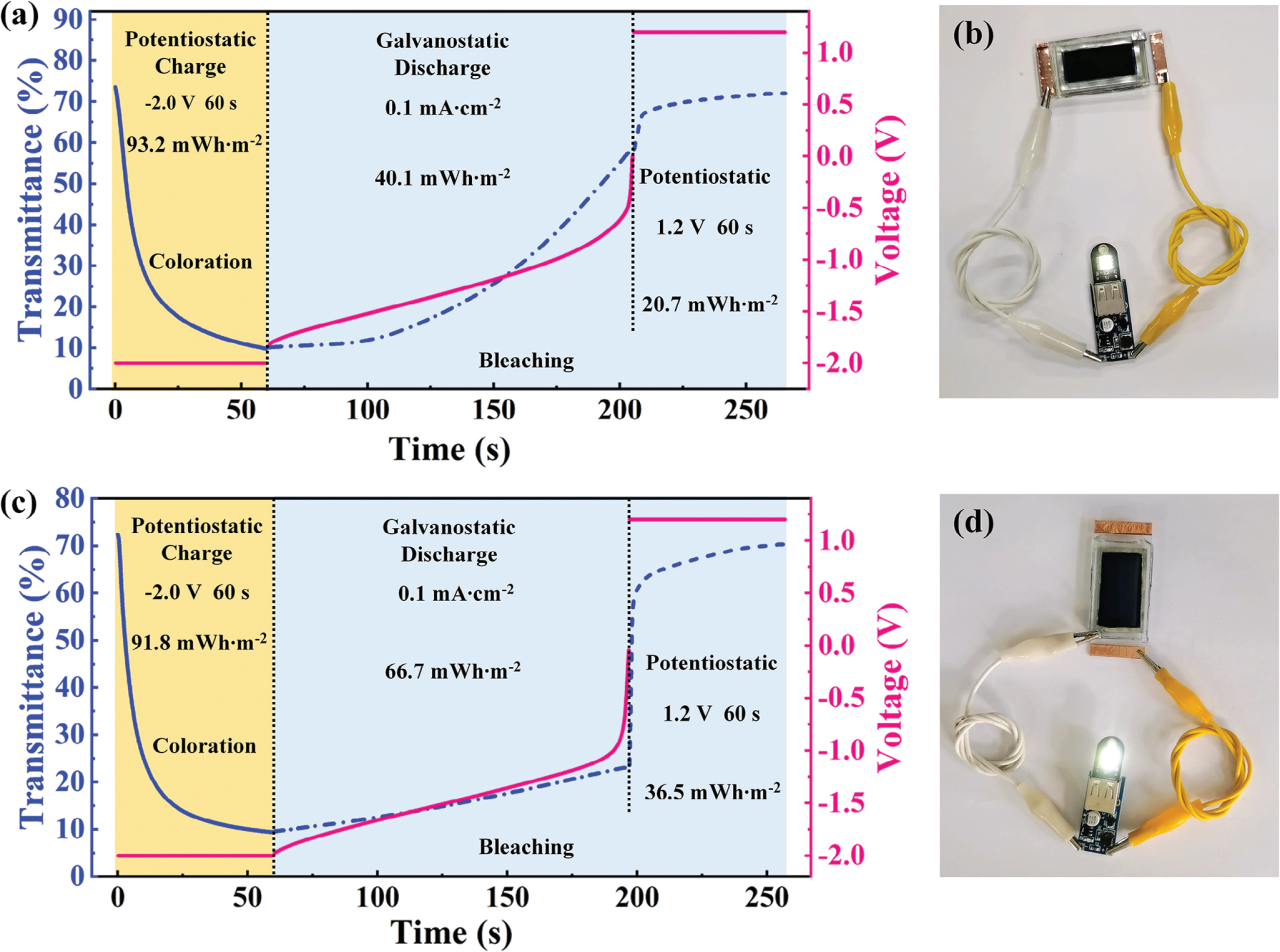
Energy retrieval functionality of the Ce_4_W_9_O_33_/Zn/NiO device. a) Potentiostatic charge at −2.0 V for 60 s, galvanostatic discharge at 0.1 mA cm^−2^, and potentiostatic charge at 1.2 V for 60 s between Ce_4_W_9_O_33_ and NiO electrodes; and the corresponding changes of the transmittance at 633 nm. b) The digital photo of a white LED powered by Ce_4_W_9_O_33_ and NiO electrodes. c) Potentiostatic charge at −2.0 V for 60 s between Ce_4_W_9_O_33_ and NiO electrodes, galvanostatic discharge at 0.1 mA cm^−2^ between Zn/NiO electrode, potentiostatic charge at 1.2 V for 60 s between Zn and Ce_4_W_9_O_33_ electrodes; and the corresponding changes of the transmittance at 633 nm. d) The digital photo of a white LED powered by Zn and NiO electrodes.

The integration of electrochromic devices with energy storage is an appealing concept for green buildings, due to their potential energy retrieval functionality. In this work, the energy consumption of the electrochromic device during its coloration and bleaching process is detailly demonstrated. The Ce_4_W_9_O_33_/NiO complementary device is potentiostatically charged at −2.0 V for 60 s (**Figure** **5**a), consuming 93.2 mWh m^−2^ of energy during coloration (the transmittance at 633 nm decreases from 73.6% to 9.8%). In the bleaching process, the Ce_4_W_9_O_33_/NiO complementary device is discharged to 0 V at 0.1 mA cm^−2^, releasing 40.1 mWh m^−2^ of energy. However, the device could not return to its fully transparent state by this process (the transmittance at 633 nm only increases to 57.6%). A voltage of 1.2 V between Ce_4_W_9_O_33_ and NiO electrodes is needed for 60 s to recover the device to its fully transparent state, consuming an additional energy of 20.7 mWh m^−2^ (Figure 5a). Therefore, the overall energy consumption of the device is up to 73.8 mWh m^−2^ (93.2 + 20.7−40.1 = 73.8 mWh m^−2^). The energy of the Ce_4_W_9_O_33_/NiO complementary device released in the discharge process is used to light up an LED (Figure 5b). Most importantly, a high potential difference (i.e., open circuit voltage) of ≈2.1 V is measured between Zn and NiO electrodes when the device is colored at −2.0 V (Figure S18, Supporting Information). Therefore, the stored energy can also be released by discharged to 0 V at 0.1 mA cm^−2^ between the Zn and NiO electrodes (Figure 5c). After that, the device can return to its fully transparent state after a potentiostatical charge process between Zn and Ce_4_W_9_O_33_ electrodes. The overall round‐trip energy consumption is reduced to 61.6 mWh m^−2^ due to the energy retrieval and self‐bleaching properties of the NiO electrode by employing the Zn anode. The energy released in this process can also be used to light up an LED (Figure 5d). The energy recycling performance of the Ce_4_W_9_O_33_/Zn/NiO device is better than the newly developed dual‐band electrochromic device.^[^ ^24^ ^]^

## 3 Conclusion

In conclusion, we have successfully demonstrated a novel Ce_4_W_9_O_33_ electrode for both electrochromic and energy storage applications for the first time. The Ce_4_W_9_O_33_ electrode exhibits a large optical modulation (74.7%) across the solar range of 400–1200 nm as well as a discharging areal capacity of 71.2 mAh m^−2^ at 0.1 mA cm^−2^. The diffusion‐controlled and surface capacitance‐controlled electrochemical reactions upon Zn^2+^ ion insertion endow the Ce_4_W_9_O_33_ electrode an excellent electrochromism in three distinct color states. The “bright and warm” state is transparent to both VIS (85.8%) and NIR (85.8%); the “dark and cool” state could effectively block almost all of the NIR (95.1%) and 80.8% of the VIS lights; while the “bright and cool” state could block 70.8% of the NIR light concurrently with a good transmittance (62.8%) at 550 nm. Moreover, we demonstrated a new class of dual‐mode electrochromic platforms by integrating zinc anode‐based and Ce_4_W_9_O_33_/NiO complementary devices. Besides the first demonstration of Zn^2+^‐triggered electrochromism in NiO electrodes, the prototype dual‐mode platform enables an added “dark and warm” state, when a voltage of 2.0 V is applied between the Zn and NiO electrodes. Such newly added optical state shows 77.3% of VIS shielding while preserving 48.2% of NIR transparency. Additionally, the device delivers a good energy retrieval functionality, which is better than previous results. Our strategy upon selective regulation of VIS and NIR sunlight/radiation paves the way for developing and constructing next‐generation electrochromic devices, making electrochromic technology more intelligent and efficient.

## 4 Experimental Section

### Materials

Sodium tungstate dihydrate (Na_2_WO_4_·2H_2_O, 98%), cerium nitrate hexahydrate (Ce(NO_3_)_3_·6H_2_O, 99.99%), sodium sulfate (Na_2_SO_4_, 99%), nickel chloride hexahydrate (NiCl_2_·6H_2_O, 98%), potassium persulfate (K_2_S_2_O_8_, 99.5%), urea (CH_4_N_2_O, 99%), and hydrochloric acid (HCl, 37%) were purchased from Shanghai Titan Scientific Co., Ltd. (China), and used without further purification. Deionized water was used throughout. Fluorine‐doped tin oxide (FTO) transparent conductive glasses (square resistance <15 Ω sq^−1^, transmittance >83%) were purchased from Zhuhai Kaivo Optoelectronic Technology Co., Ltd. (China).

### Growth of Ce_4_W_9_O_33_ Film

The Ce_4_W_9_O_33_ films were successfully grown on FTO glass through a typical hydrothermal method. The precursor solution for hydrothermal use was made by dissolving Na_2_WO_4_·2H_2_O, Na_2_SO_4_, and Ce(NO_3_)_3_·6H_2_O in 40 mL of deionized water under magnetic stirring. Then a mixed solution of 25 mmol L^−1^ of Na_2_WO_4_·2H_2_O, 25 mmol L^−1^ of Na_2_SO_4_ and 12.5 mmol L^−1^ of Ce(NO_3_)_3_·6H_2_O was obtained. After adding 0.8 mL of HCl, the resulting solution was transferred into a 50 mL Teflon‐lined stainless‐steel autoclave. A piece of cleaned FTO glass was placed into the autoclave and leaned on the wall with the conductive side facing down. The autoclave was subsequently sealed and maintained at 180°C for 3 h. Finally, the Ce_4_W_9_O_33_ film‐coated FTO glass was taken out, rinsed with deionized water, and dried naturally. Although the electrode films fabricated via hydrothermal methods suffer a big challenge in scalability, the current work reported that the Ce_4_W_9_O_33_ electrode material was capable of regulating visible light and near‐infrared light on demand for the first time. In addition, Ce_4_W_9_O_33_ powder materials can be synthesized via the same approach, indicating that post‐modification of the Ce_4_W_9_O_33_ powder materials for solution‐processable inks may be a proper strategy for fabricating large‐area, high‐quality electrodes.

### Preparation of NiO Film

Porous NiO films were also grown on FTO glasses through a hydrothermal method. First, a mixed solution of 20 mmol L^−1^ of NiCl_2_·6H_2_O and 12 mmol L^−1^ of K_2_S_2_O_8_ was prepared. Then 3 g of urea was added under magnetic stirring, and the resulting solution was transferred into a 50 mL Teflon‐lined stainless‐steel autoclave. A piece of cleaned FTO glass was placed into the autoclave, which was sealed and maintained at 110°C for 90 min.

### Fabrication of Ce_4_W_9_O_33_/Zn/NiO Device

The Ce_4_W_9_O_33_/Zn/NiO device was fabricated by sandwiching a zinc frame within the Ce_4_W_9_O_33_ electrode and NiO electrode and sealed with a silicone double‐sided adhesive tape. Finally, 0.5 mol L^−1^ of ZnSO_4_ aqueous solution was injected into the space of the device and used as the electrolyte.

### Characterizations

X‐ray diffraction (XRD) technique using a D8‐ADVANCE (Bruker, Germany, Cu Kα (λ = 0.15418 nm) with radiation at 40 kV and 200 mA in the 2θ range of 10−80°), field‐emission scanning electron microscope (FESEM, ZEISS Gemini SEM 300, Germany), Scanning transmission electron microscope/transmission electron microscope (STEM/TEM, JEOL JEM 2100F, Japan) and X‐ray photoelectron spectroscopy (XPS) (Thermo Scientific K‐Alpha, USA) using Al Kα radiation (hν = 1486.6 eV) were used to identify the morphology, structure, and composition of the Ce_4_W_9_O_33_ film. Electrochemical measurements of the Ce_4_W_9_O_33_ film such as cyclic voltammetry (CV) curves, charging/discharging curves, and cyclic stability at different current densities were recorded by an electrochemical workstation (Gamry, Interface 1010E, USA) in a three‐electrode system, which consisted of the Ce_4_W_9_O_33_ working electrode, a Pt sheet counter electrode, and Ag/AgCl reference electrode. 0.5 mol L^−1^ of ZnSO_4_ aqueous solution was used as electrolyte. Spectroscopy testing was conducted using a UV/Visible/Near Infrared Spectrometer (Hitachi, UH‐5700, Japan). The spectra were all tested without background correction, and the background of the FTO glass was not deducted. The in situ transmittance spectra, switching times of the as‐grown Ce_4_W_9_O_33_ film and Ce_4_W_9_O_33_/Zn/NiO device at different color states were recorded for calculating the optical modulation, solar irradiance transmittance and modulation, switching times, and coloration efficiency. The in situ transmittance spectra of the as‐grown Ce_4_W_9_O_33_ film were measured through a three‐electrode cell (consisting of the Ce_4_W_9_O_33_ working electrode, a Pt sheet counter electrode, an Ag/AgCl reference electrode, and 0.5 mol L^−1^ of ZnSO_4_ aqueous solution). The data over the solar spectrum range of 400–1200 nm were recorded from a UV/Visible/Near Infrared Spectrometer (UH‐5700) coupled with the electrochemical workstation. The optical modulation was calculated from the following formulae: Δ*T* = *T*_b_–*T*_c_, where *T*_b_ and *T*_c_ are the transmittance of the film in its bleached and colored states, respectively. The switching times were defined as the times required for 90% of change in the full optical modulation at the specified wavelengths. Coloration efficiency was calculated from the following formulae: CE = Δ*OD*/(*Q*/A); Δ*OD* = log(*T*_b_/*T*_c_), where *Q*/A is the injected charge *Q* per unit electrode area A. *T*_b_ and *T*_c_ are the transmittances of the films or devices in their bleached and colored states at the specified wavelengths, respectively.

## Conflict of Interest

The authors declare no conflict of interest.

## Supporting information

Supporting Information

## Data Availability

The data that support the findings of this study are available from the corresponding author upon reasonable request.
