# Supplementary material for: Quadruple Control Electrochromic Devices Utilizing Ce4W9O33 Electrodes for Visible and Near‐Infrared Transmission Intelligent Modulation
Source: Adv Sci (Weinh). 2024 Feb 4;11(14):2307223. doi: 10.1002/advs.202307223 (PMC11005709; doi:10.1002/advs.202307223)
Supplement: Supplementary file 1 — Supporting Information [file ADVS-11-2307223-s001.pdf]

ADVANCED SCIENCE
Open Access

Supporting Information

for *Adv. Sci.*, DOI 10.1002/advs.202307223

Quadruple Control Electrochromic Devices Utilizing $Ce_4W_9O_{33}$ Electrodes for Visible and Near-Infrared Transmission Intelligent Modulation

Dongyun Ma, Ting Yang, Xingzhe Feng, Pengfei Wang, Jiahui Huang, Jinmin Wang and Haizeng Li**

Supporting Information

Quadruple Control Electrochromic Devices Utilizing $Ce_4W_9O_{33}$ Electrodes for Visible and Near-Infrared Transmission Intelligent Modulation

Dongyun Ma, Ting Yang, Xingzhe Feng, Pengfei Wang, Jiahui Huang, Jinmin Wang, and Haizeng Li**

D. Ma, T. Yang, X. Feng, P. Wang, J. Huang, J. Wang
School of Materials and Chemistry, University of Shanghai for Science and Technology, Shanghai 200093, China.
E-mail: jmwang@usst.edu.cn

H. Li
Optics and Thermal Radiation Research Center, Institute of Frontier & Interdisciplinary Science, Shandong University, Qingdao, Shandong, 266237, China
Email: haizeng@sdu.edu.cn

Supplementary figures and comments

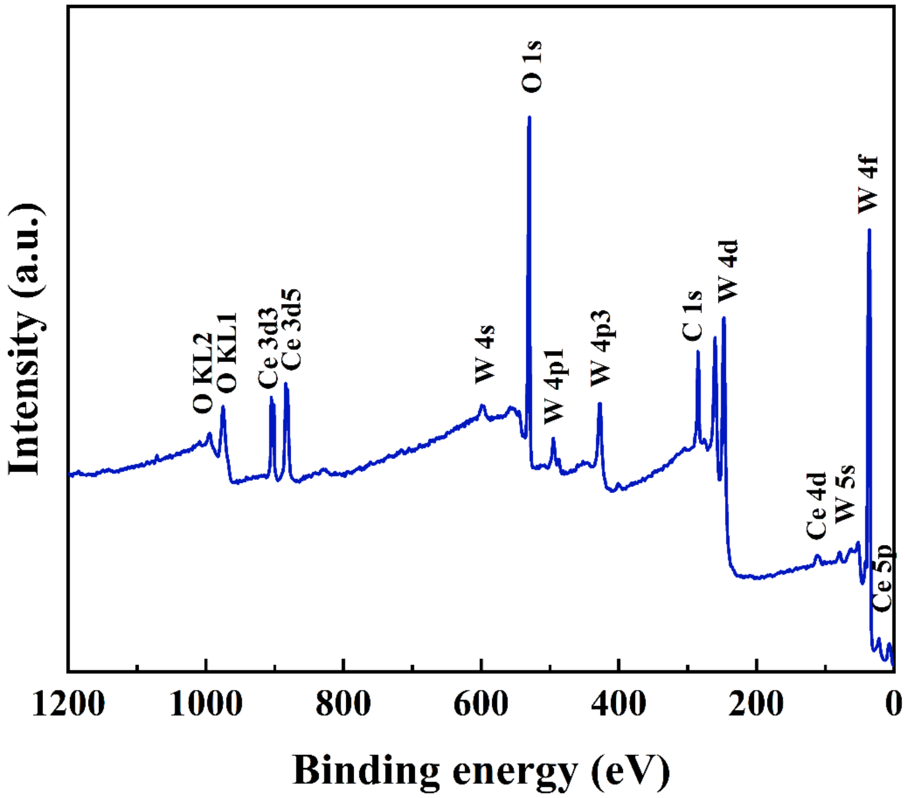

Figure S1. XPS survey spectrum of the as-grown $Ce_4W_9O_{33}$ film.

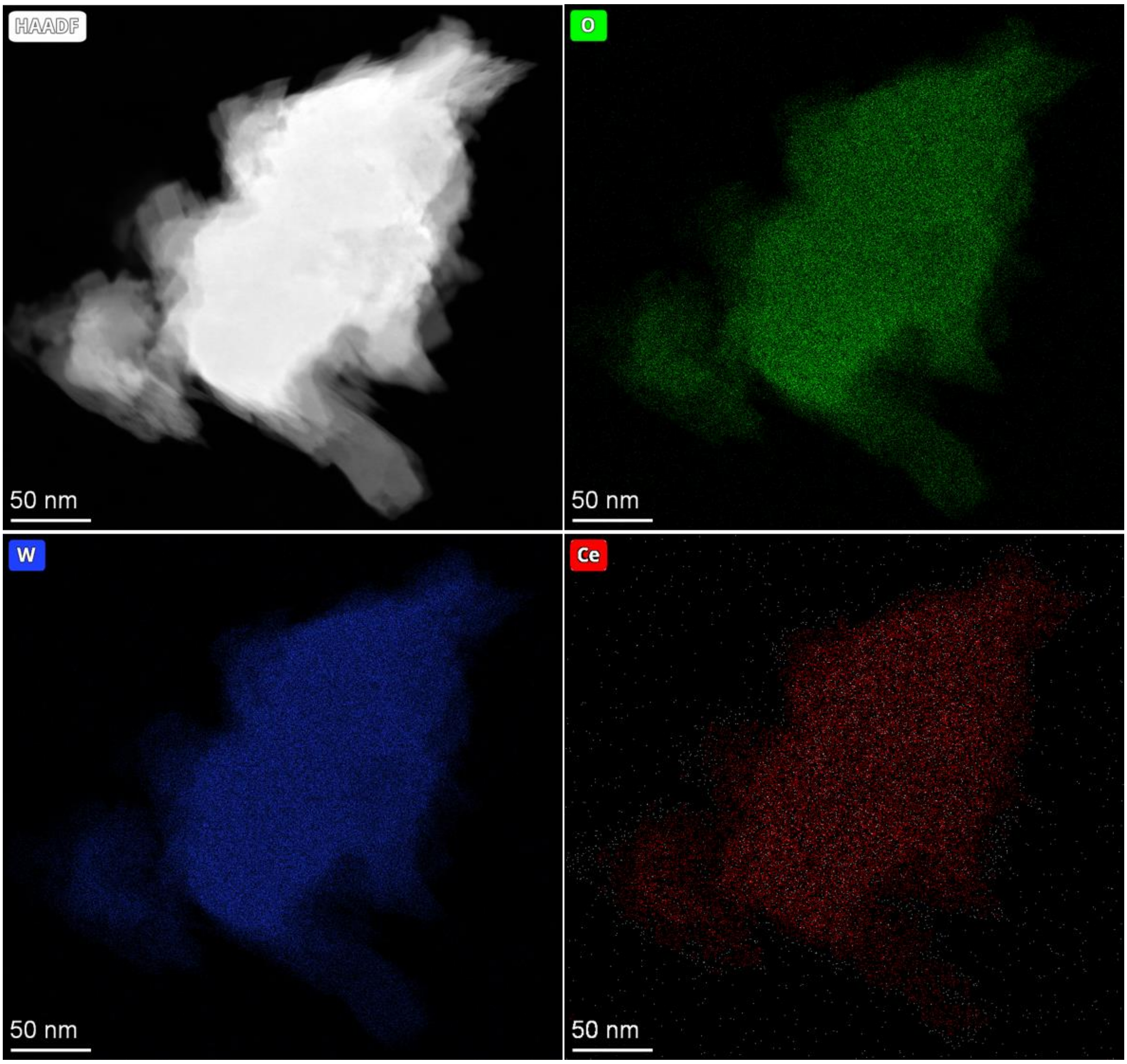

Figure S2. STEM-EDS elemental mapping results for the $Ce_4W_9O_{33}$ film.

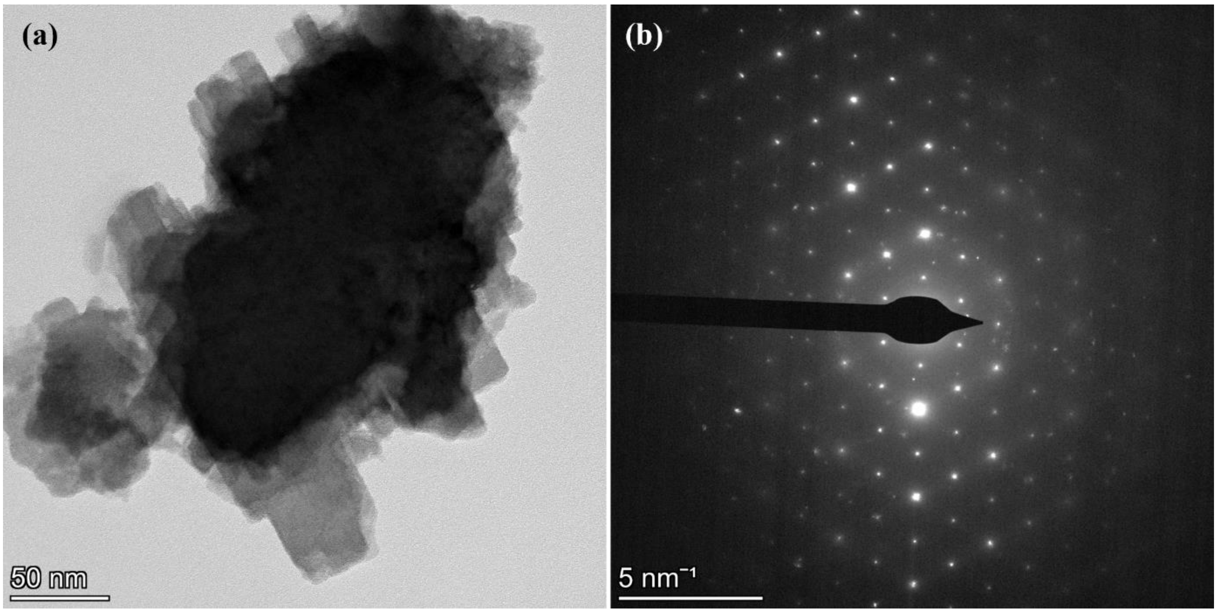

Figure S3. a) TEM image and b) selected-area electron diffraction (SAED) patterns of the $Ce_4W_9O_{33}$ film.

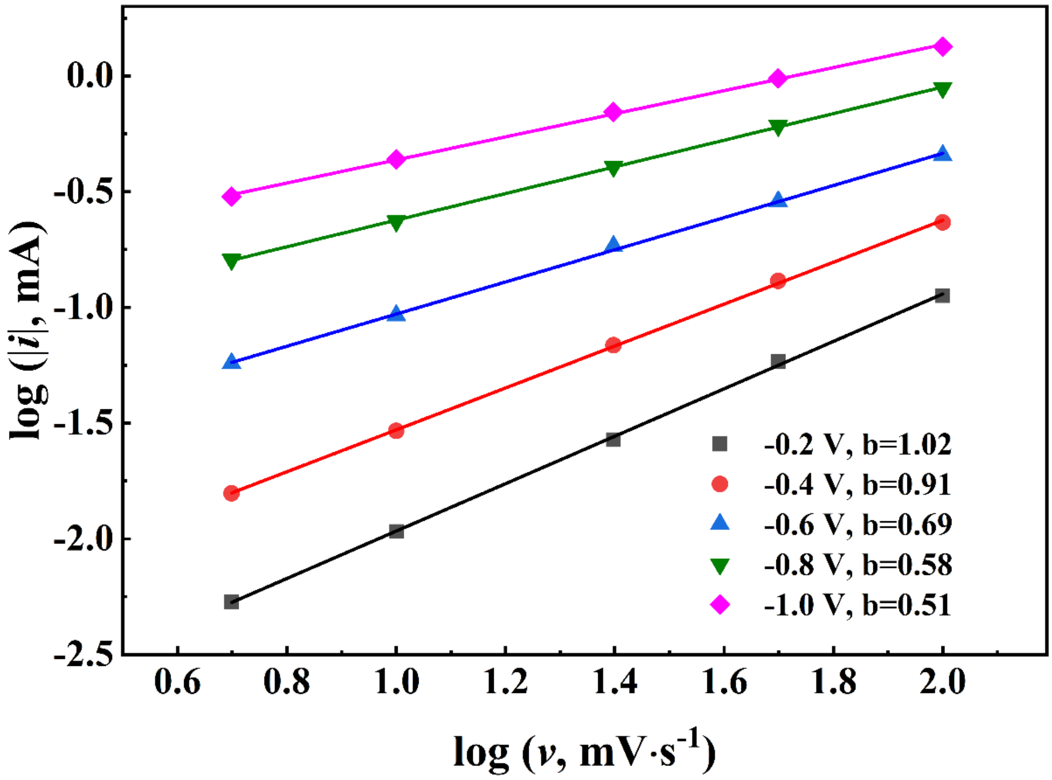

Figure S4. Plots of log*i* *vs*. log*v* for the $Ce_4W_9O_{33}$ electrode at potentials of -0.2, -0.4, -0.6, -0.8 and -1.0 V, respectively.

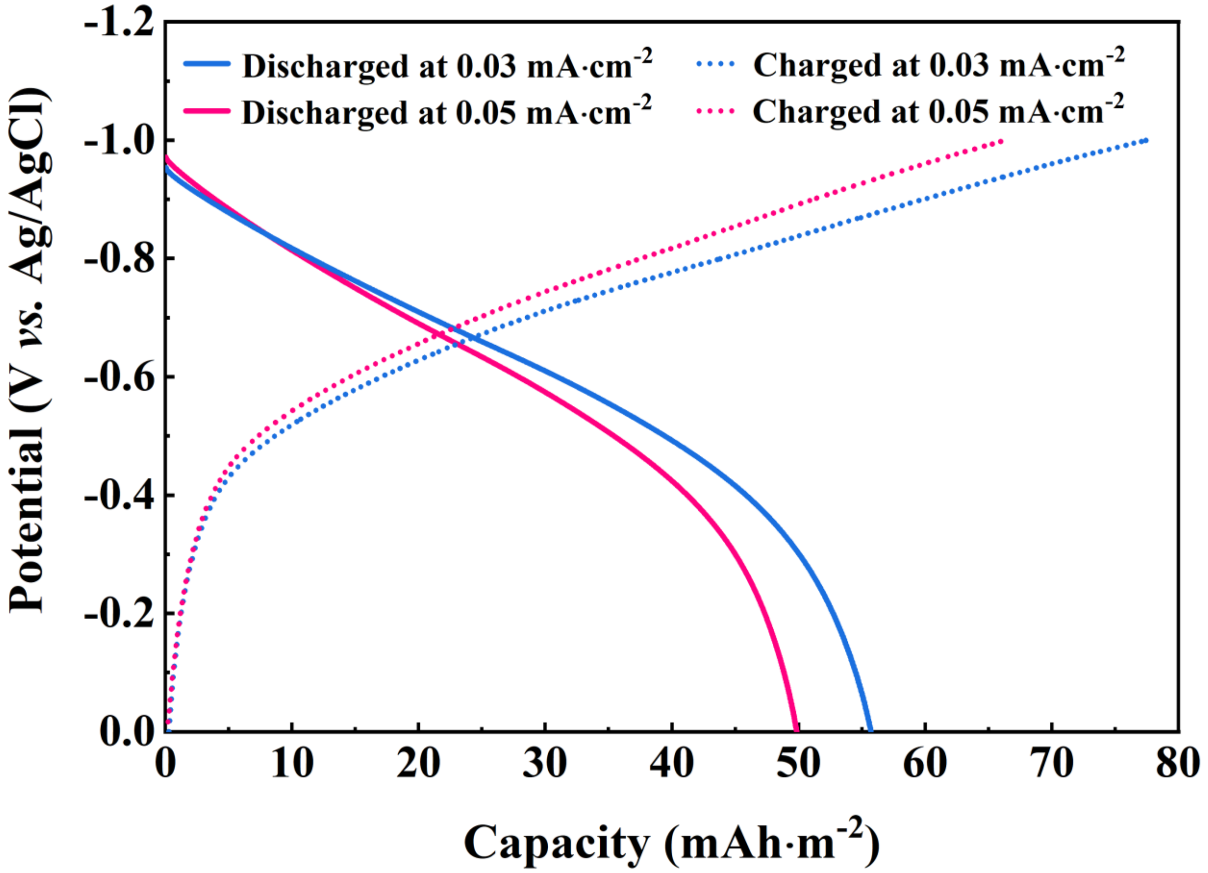

Figure S5. GCD curves of the as-prepared $Ce_4W_9O_{33}$ film electrode at current densities of 0.03 and 0.05 mA·cm^{-2}, respectively.

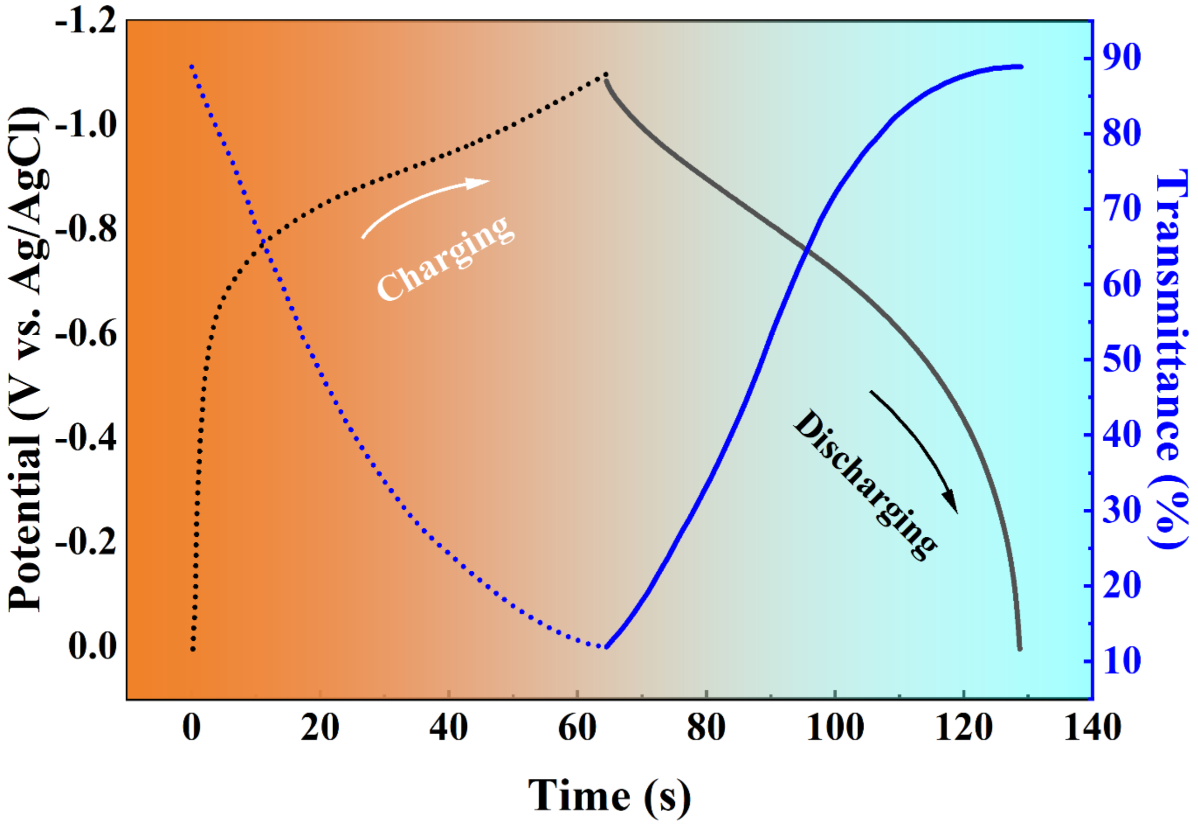

Figure S6. GCD profiles and the corresponding *in situ* transmittance change curve at 633 nm for the $Ce_4W_9O_{33}$ film electrode at 0.3 mA·cm^{-2}.

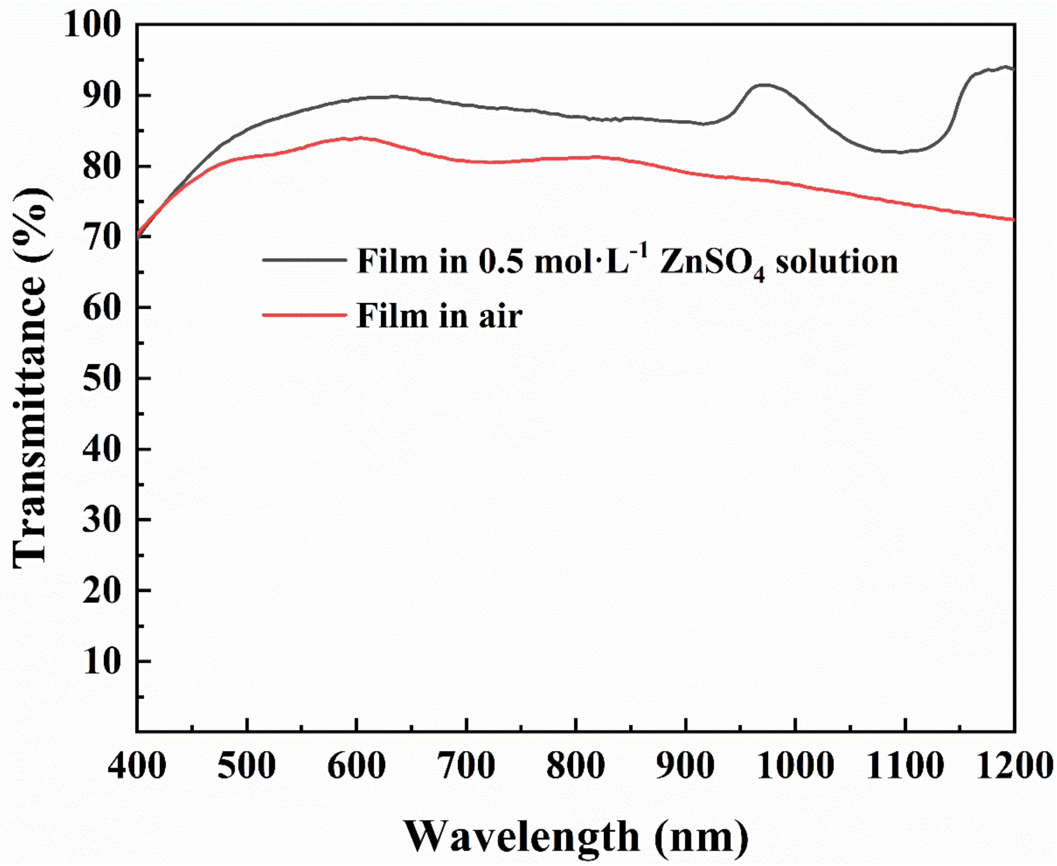

Figure S7. Transmittance spectra of the $Ce_4W_9O_{33}$ film tested in air and electrolyte solution (i.e., 0.5 M $ZnSO_4$ solution).

As shown in **Figure S7**, the transmittance of $Ce_4W_9O_{33}$ film measured in air is lower than that measured in a 0.5 M $ZnSO_4$ solution. This result indicates that the use of electrolyte enables a reduced haze effect of the $Ce_4W_9O_{33}$ film.

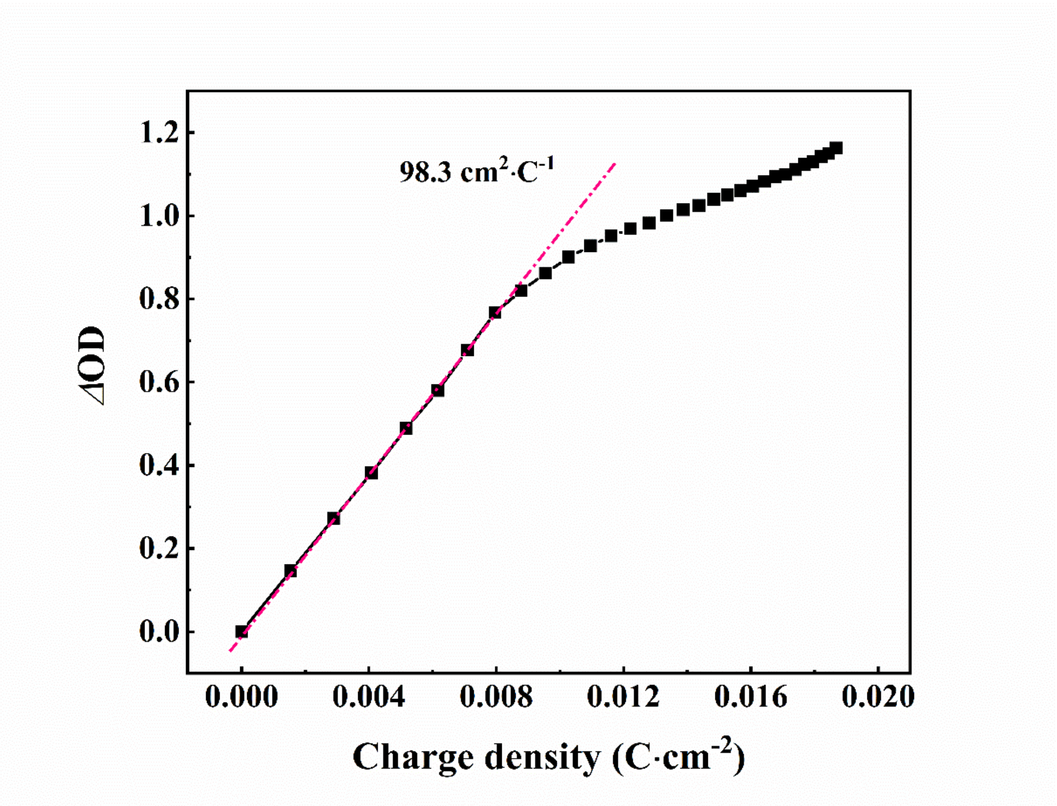

Figure S8. Optical density variations with respect to the charge density for the $Ce_4W_9O_{33}$ film measured at 1200 nm.

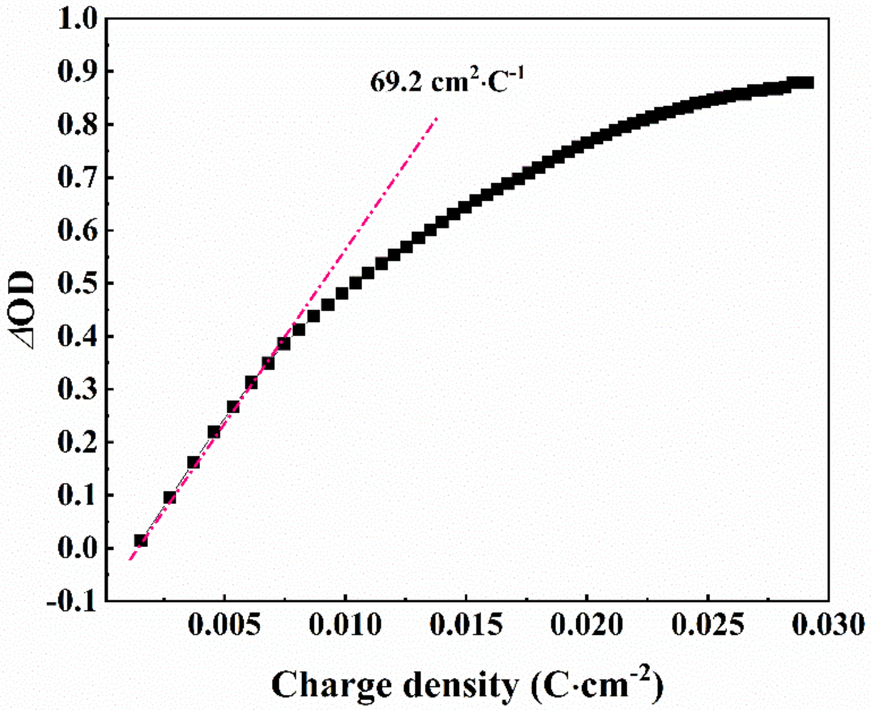

Figure S9. Optical density variations with respect to the charge density for the $Ce_4W_9O_{33}$ film measured at 633 nm.

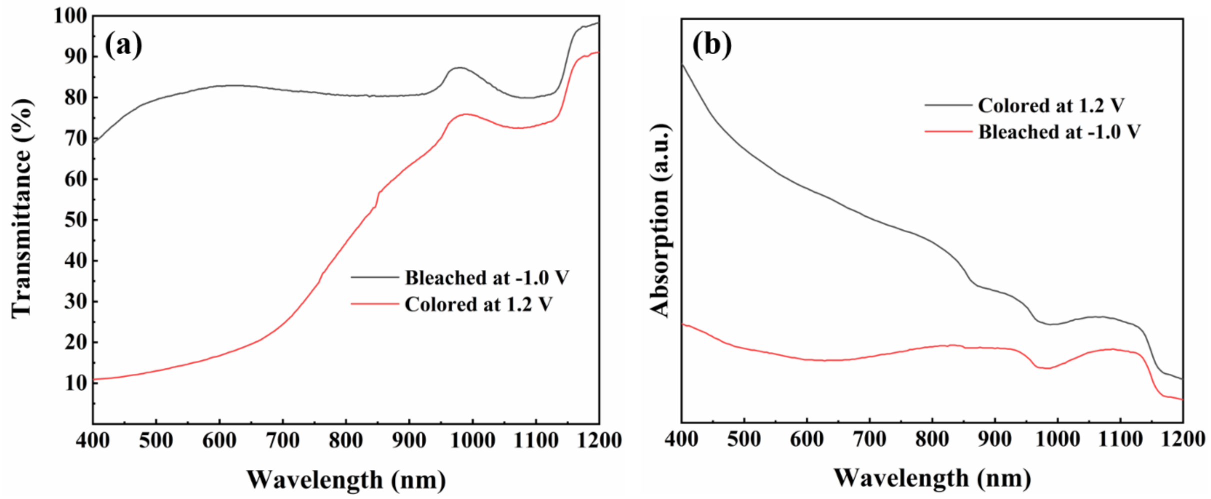

Figure S10. a) Transmittance spectra and b) absorption spectra of the hydrothermally grown NiO film in its colored and bleached states.

The hydrothermally grown NiO film shows a large optical modulation in the VIS region (66.6% at 550 nm) while maintaining a high NIR transmission. The average optical modulation is calculated to be 60.0% in the VIS range of 400-800 nm. Different from n-type WO_3-based plasmonic electrochromic materials, p-type NiO has no local surface plasmon resonance (LSPR) effect to regulate NIR light. The large optical modulation in the VIS region and high NIR transmission of NiO film is attributed to its low VIS transmittance and high NIR transmittance in the colored state. This stems from the strong VIS absorption and weak NIR absorption of the colored NiO film.[1-3]

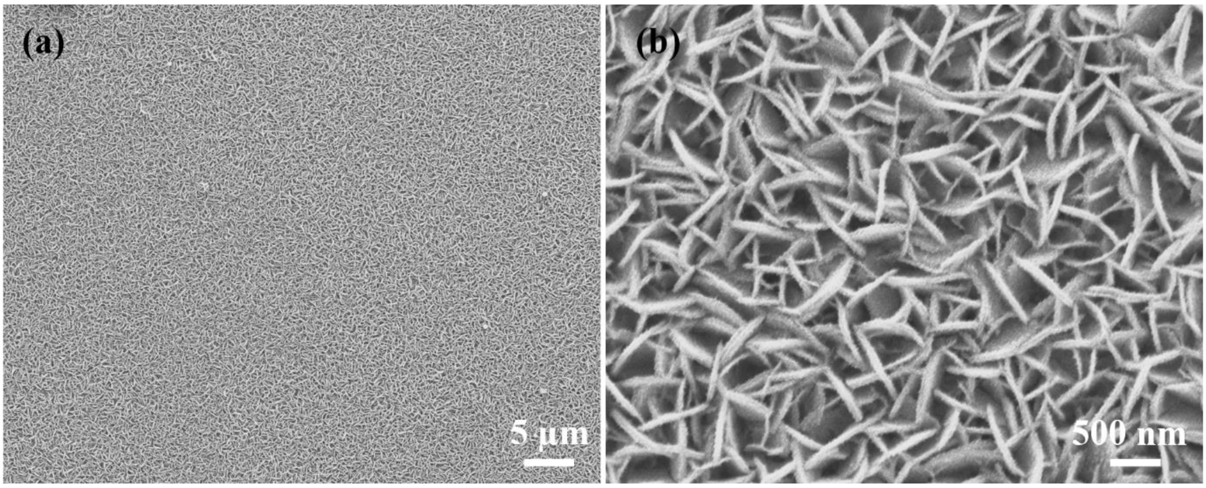

Figure S11. SEM images for the hydrothermally grown NiO film.

The porous NiO film was directly grown onto FTO glass by a simple hydrothermal method. The growth mechanism of NiO is similar to that of the $Ce_4W_9O_{33}$ film. The difference is that a weakly alkaline environment was formed due to the addition of urea in the precursor solution. Thus, $Ni(OH)_2$ nanocrystals were probably first formed and subsequently decomposed to NiO under hydrothermal temperature and pressure. Figure S11 shows that the FTO surface is uniformly covered by the NiO film, which is composed of NiO nanoflakes with a thickness of about 50-60 nm.

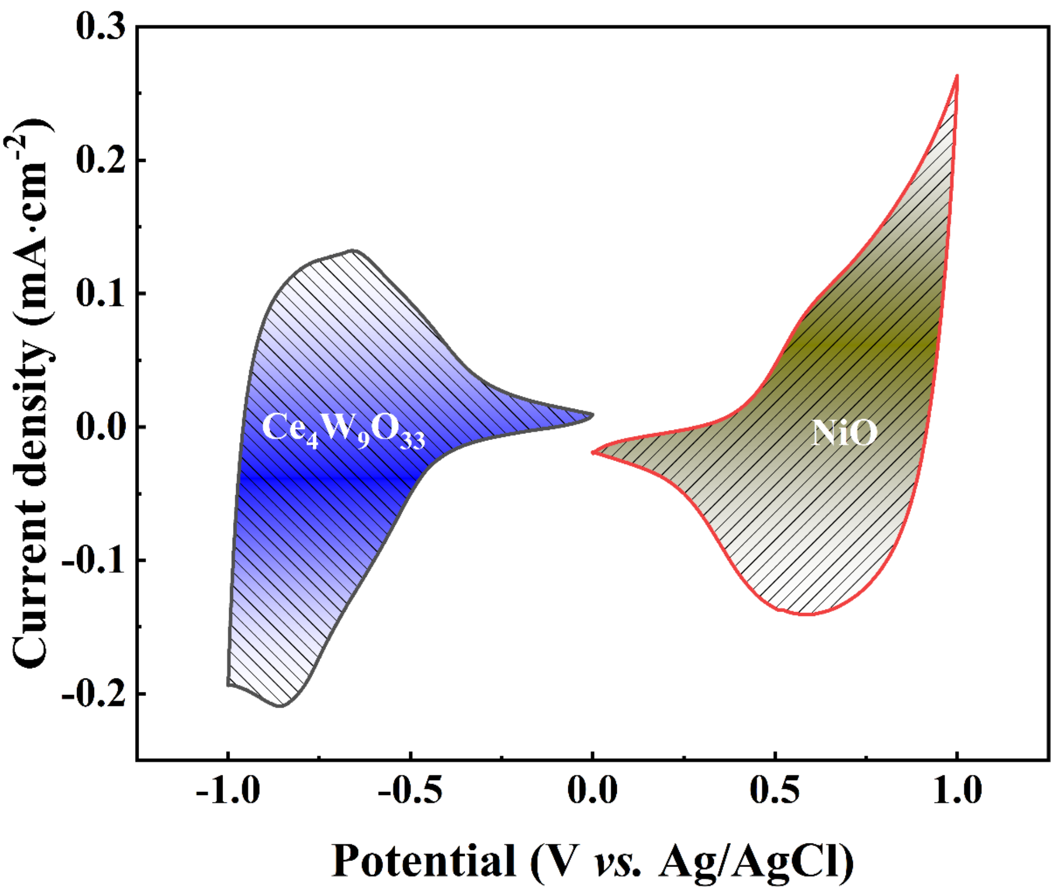

Figure S12. CV curves of the $Ce_4W_9O_{33}$ and NiO films, measured at a scan rate of 5 $mV{\cdot}s^{-1}$.

To construct highly efficient complementary electrochromic devices, it is necessary to achieve matched charge storage capabilities and electrochromic behaviors between anode and cathode electrodes. As shown in Figure S12, the $Ce_4W_9O_{33}$ film electrode delivers a charge storage capacity of 0.147 $mW{\cdot}cm^{-2}$, which matches well with that of the NiO film electrode (0.143 $mW{\cdot}cm^{-2}$).

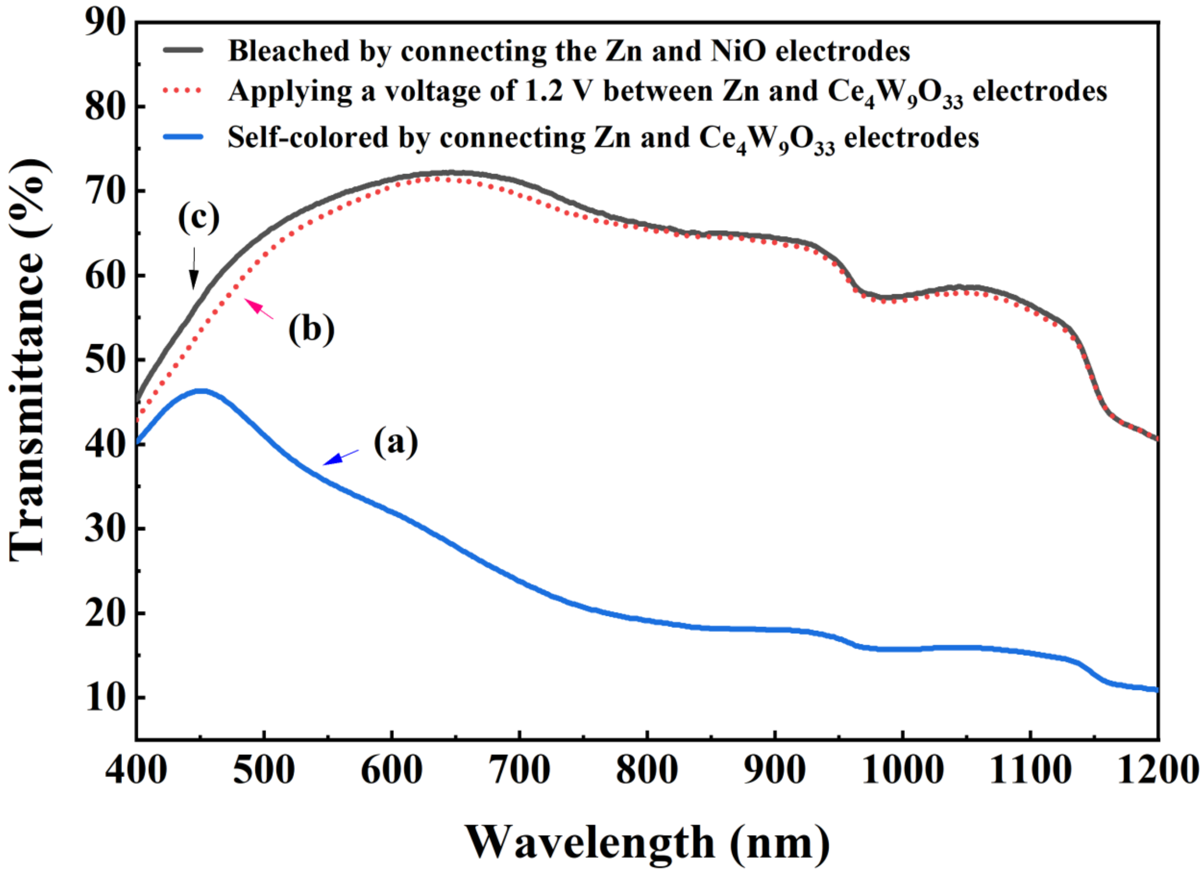

Figure S13. Transmittance spectra of the $Ce_4W_9O_{33}$/Zn/NiO device at the following states: a) self-colored by connecting the Zn and $Ce_4W_9O_{33}$ electrodes. b) Bleached by applying a voltage of 1.2 V between the Zn and $Ce_4W_9O_{33}$ electrodes in the "bright and cool" state. c) Self-bleached by connecting the Zn and NiO electrodes at the "dark and warm" state.

As an active metal, Zn can be easily oxidized by releasing electrons to form Zn^{2+} ions, while W^{6+} can be reduced to W^{5+} through accepting electrons. Therefore, the $Ce_4W_9O_{33}$ electrode can be self-colored by connecting the Zn and $Ce_4W_9O_{33}$ electrodes (Figure S13a). The device can switch to the "bight and warm" state after applying a voltage of 1.2 V between the Zn and $Ce_4W_9O_{33}$ electrodes (Figure S13b). Additionally, the "dark and warm" state can also return to the "bight and warm" state through the self-bleaching of NiO electrode by connecting it to Zn electrode (Figure S13c).

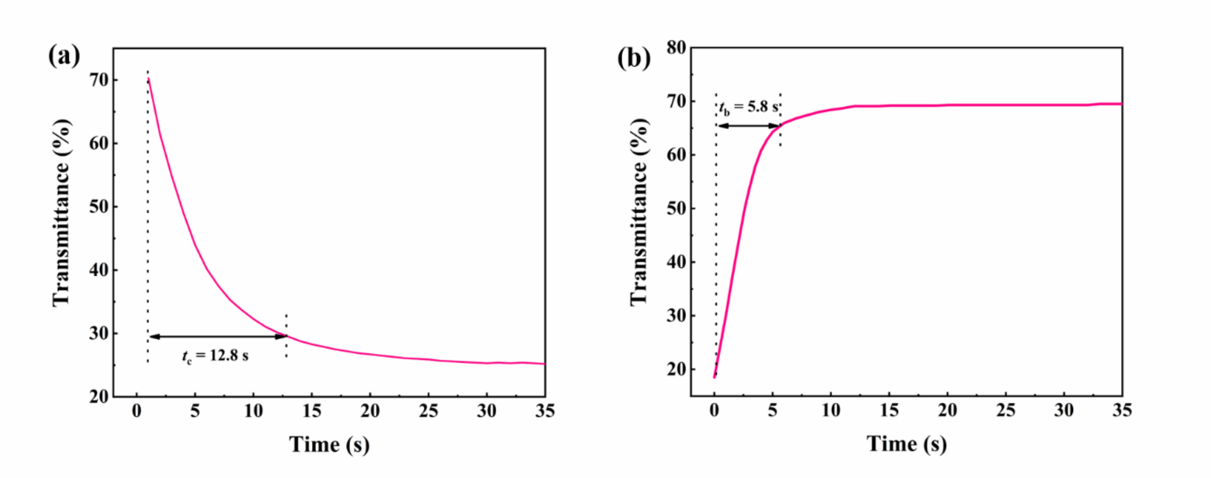

Figure S14. Real-time transmittance changes of the $Ce_4W_9O_{33}$/Zn/NiO device at the following states: a) Self-colored by connecting the Zn and $Ce_4W_9O_{33}$ electrodes. b) Self-bleached by connecting the Zn and NiO electrodes at the "dark and warm" state.

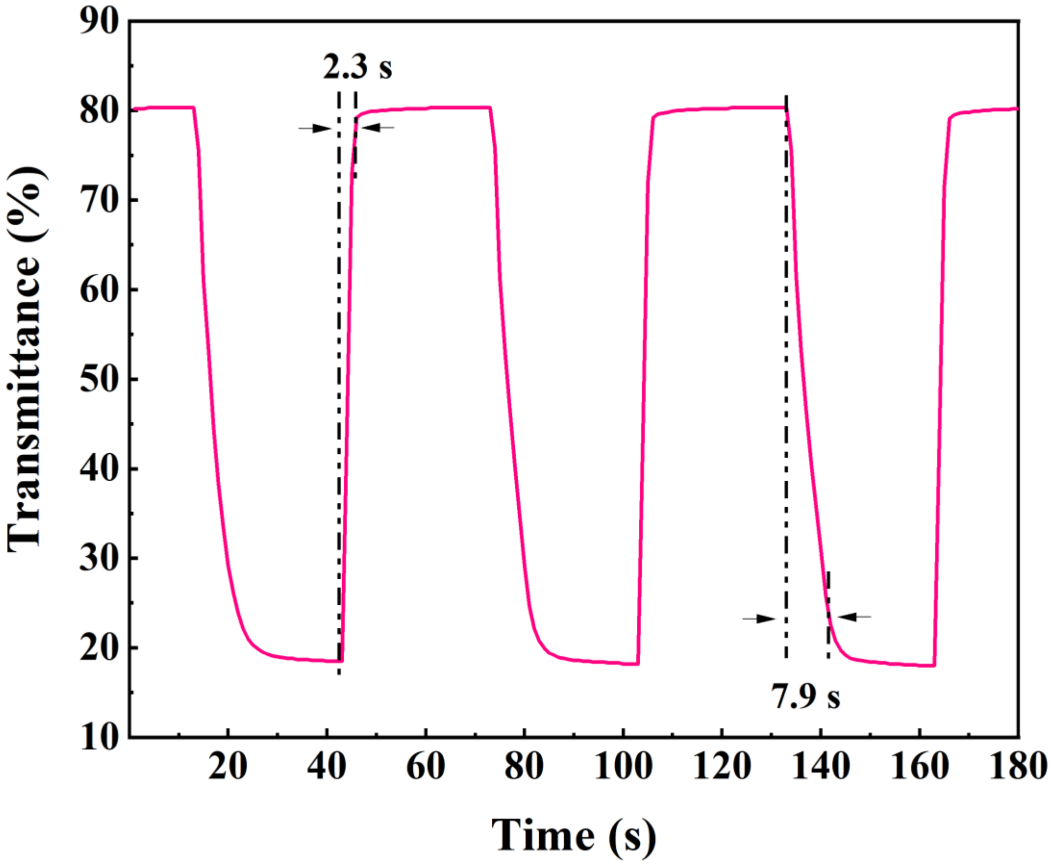

Figure S15. Real-time transmittance changes during reversible switching of the NiO film measured at 550 nm.

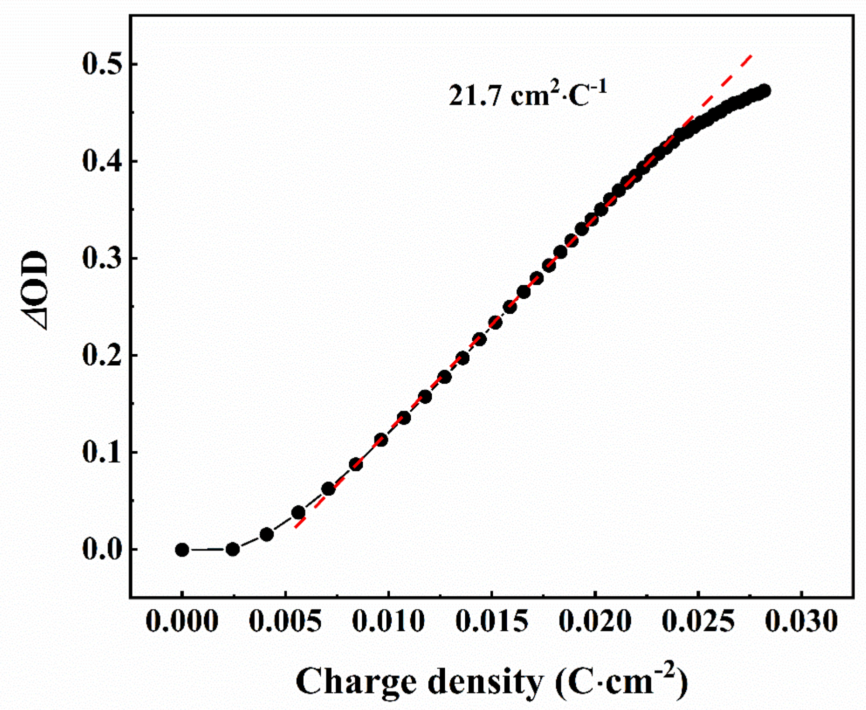

Figure S16. Optical density variations with respect to the charge density of the NiO film recorded at 550 nm.

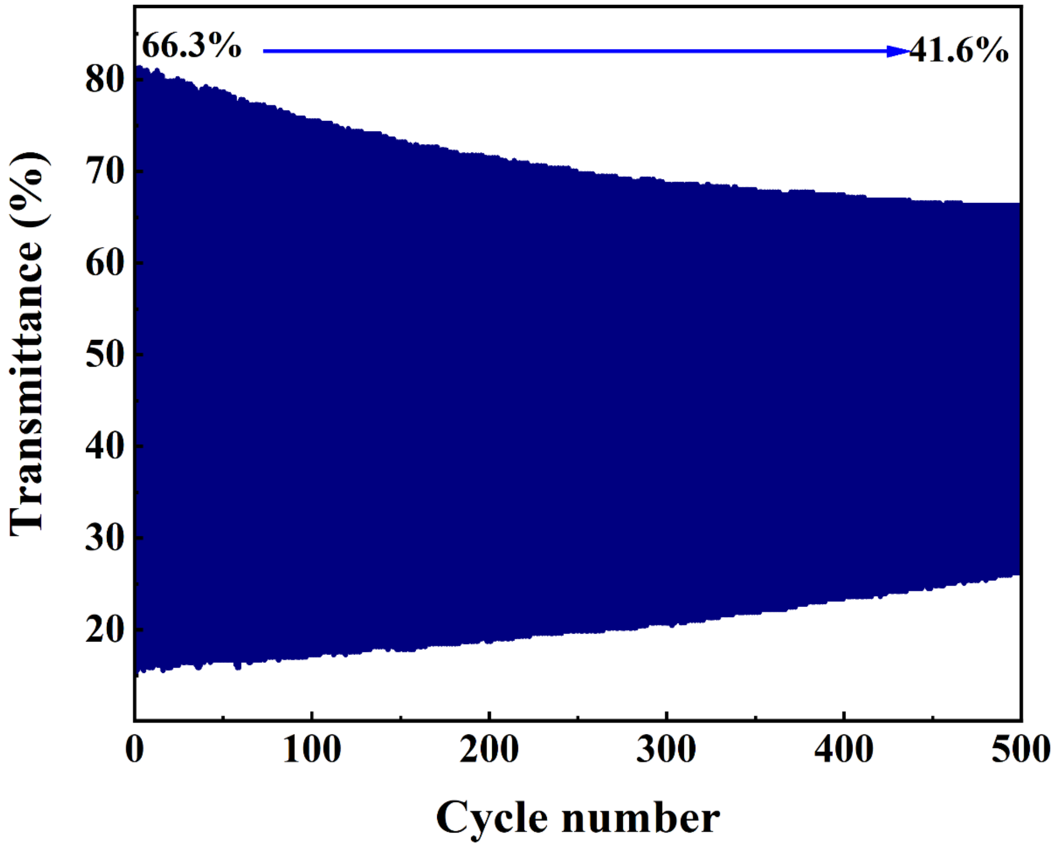

Figure S17. Cycle performance of the NiO film measured at 550 nm.

The NiO film exhibits a poor cycle stability, where the optical modulation decreases from 66.3% to 41.6%, maintaining only 62.7% of its initial value after 500 cycles.

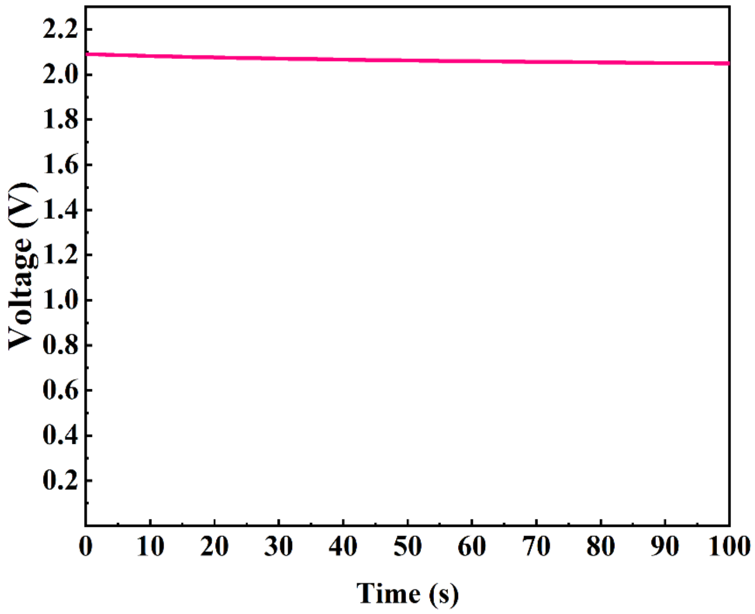

Figure S18. Open circuit voltage of the full charged device measured between the Zn and NiO electrodes.

References

[1] K. Sun, F. H. Saadi, M. F. Lichterman, W. G. Hale, H.-P. Wang, X. Zhou, N. T. Plymale, S. T. Omelchenko, Jr-H. He, K. M. Papadantonakis, B. S. Brunschwig, N. S. Lewis, *Proc. Natl. Acad. Sci*. **2015**, *112*, 3612.

[2] K. Sun, M. T. McDowell, A. C. Nielander, S. Hu, M. R. Shaner, F. Yang, B. S. Brunschwig, N. S. Lewis, *J. Phys. Chem. Lett*. **2015**, *6*, 592.

[3] J. Wang, R. Zhu, Y. Gao, Y. Jia, G. Cai, *J. Phys. Chem. Lett*. **2023**, *14*, 2284.
